# Crystal structure of aqua­tris­(isonicotinamide-κ*N*)bis­(thio­cyanato-κ*N*)cobalt(II) 2.5-hydrate

**DOI:** 10.1107/S2056989016012470

**Published:** 2016-08-09

**Authors:** Tristan Neumann, Inke Jess, Christian Näther

**Affiliations:** a Institut für Anorganische Chemie, Christian-Albrechts-Universität Kiel, Max-Eyth Strasse 2, D-24118 Kiel, Germany

**Keywords:** crystal structure, discrete complex, cobalt(II) thio­cyanate, isonicotinamide, hydrogen bonding

## Abstract

The asymmetric unit of the title compound, [Co(NCS)_2_(C_6_H_6_N_2_O)_3_(H_2_O)]·2.5H_2_O, comprises one Co^II^ cation, three isonicotinamide ligands, two thio­cyanate anions, one aqua ligand and two water solvent mol­ecules in general positions, as well as one water solvent mol­ecule that is located on a twofold rotation axis. The Co^II^ cations are octa­hedrally coordinated by two terminally *N*-bonded thio­cyanate anions, one water mol­ecule and three isonicotinamide ligands, each coordinating *via* the pyridine N atom. The discrete complexes are linked by inter­molecular O—H⋯O, N—H⋯O and N—H⋯S hydrogen bonding into a three-dimensional network that contains cavities in which the solvent water mol­ecules are located. The latter are linked by further O—H⋯O hydrogen bonds to the network. There are additional short contacts present in the crystal, indicative of weak C—H⋯S, C—H⋯O and C—H⋯N inter­actions.

## Abstract 2

In the crystal structure of the title compound, the Co^II^ cations are coordinated by two terminal *N*-bonded thio­cyanate anions, three isonicotinamide ligands and one water mol­ecule into discrete octa­hedral complexes that are connected by classical and non-classical hydrogen bonding into a three-dimensional network.

## Chemical context   

The synthesis of new coordination polymers with cooperative magnetic properties is still a major field in coordination chemistry. In this context, compounds that show a slow relaxation of the magnetization, such as, for example, single chain magnets, are of special inter­est because of their potential for future applications (Dhers *et al.*, 2015 ▸; Caneschi *et al.*, 2001 ▸; Liu *et al.*, 2010 ▸). To trigger such behavior, cations of large magnetic anisotropy, such as, for example, Mn^II^, Fe^II^ or Co^II^, must be linked by ligands into chains that can mediate a magnetic exchange. Therefore, we are generally inter­ested in the synthesis and the magnetic properties of Co- and Fe-containing thio- and seleno­cyanate coordination polymers (Werner *et al.*, 2014 ▸, 2015*a* ▸,*b* ▸,*c* ▸; Boeckmann *et al.*, 2012 ▸; Wöhlert *et al.*, 2014 ▸). This also includes the synthesis of discrete complexes with a terminal coordination because such compounds can be transformed into the desired polymeric compounds by thermal decomposition reactions (Näther *et al.*, 2013 ▸). In the course of our investigations, we attempted to prepare Co-containing thio­cyanate coordination compounds with isonicotinamide as ligand and obtained crystals of the title compound, [Co(NCS)_2_(C_6_H_6_N_2_O)_3_(H_2_O)]·2.5H_2_O. However, this phase could not be prepared as a pure phase. To identify these crystals, a single crystal structure analysis was performed and the results are reported herein.

## Structural commentary   

**Figure 1:**
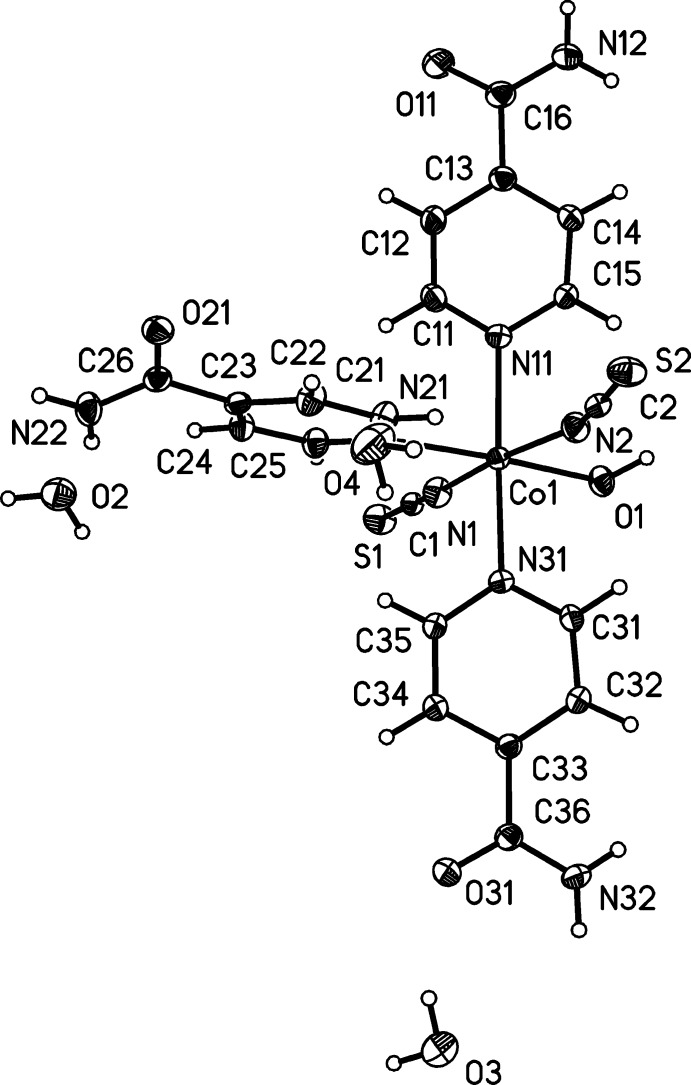
View of the asymmetric unit of the title compound with labeling and displacement ellipsoids drawn at the 50% probability level.

The asymmetric unit comprises one cobalt(II) cation, two thio­cyanate anions, three isonicotinamide ligands and three water mol­ecules (one as a ligand and two as solvent mol­ecules) that occupy general positions as well as one water solvent mol­ecule that is located on a twofold rotation axis (Fig. 1 ▸). The Co^II^ cation is coordinated by one water mol­ecule, two terminal *N*-bonded thio­cyanate anions and three terminal isonicotinamide ligands bonded through the pyridine N atom. The Co—N distances to the negatively charged anionic ligands of 2.0746 (1) and 2.0834 (17) Å are shorter than that to the neutral isonicotinamide ligands [Co—N: 2.1725 (16)–2.2059 (15) Å]. The bond angles around the Co^II^ atom deviate slightly from the ideal values [*cis* angles: 85.81 (6)–92.60 (7)°; *trans* angles: 173.17 (7)–177.74 (6)°]. The resulting coordination polyhedron can be described as a slightly distorted octa­hedron (Fig. 1 ▸)
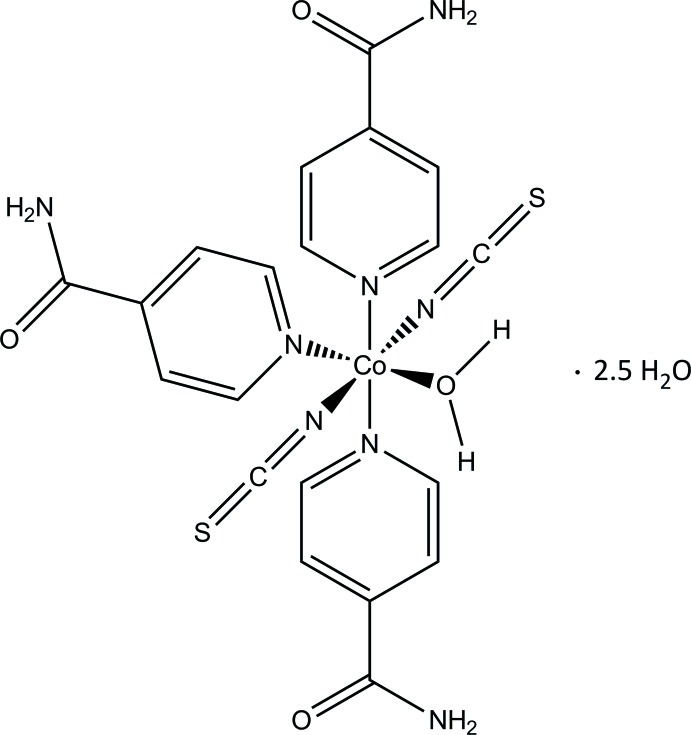
.

## Supra­molecular features   

**Figure 2:**
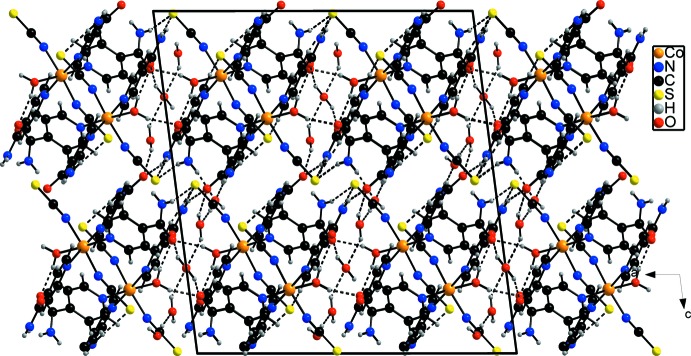
The packing in the crystal structure of the title compound in a view along the *b* axis. Inter­molecular hydrogen bonding is shown as dashed lines.

In the crystal structure, four symmetry-related complexes are linked by inter­molecular O—H⋯O hydrogen bonding between the water H atoms of the coordinating water mol­ecules of two complexes and the carbonyl O acceptor atoms of two additional complexes into eight-membered rings that are located on centres of inversion (Fig. 2 ▸). These tetra­mers are further connected by inter­molecular O—H⋯O and N—H⋯O hydrogen bonding between water mol­ecules and amide H atoms, respectively, and the carbonyl as well as water acceptor-O atoms into a three-dimensional network (Fig. 2 ▸). There are additional hydrogen bonds between the amide H atoms and the S atoms of the anionic ligands. The N—H⋯S angles deviate only slightly from 180°. Within this network cavities are formed, in which additional water mol­ecules are embedded. These solvent mol­ecules are linked by (water)O—H⋯O(water) hydrogen bonding into chain-like aggregates that consist of five water mol­ecules each, whereby the aggregates are located on twofold rotation axes. These water aggregates are linked by additional O—H⋯O hydrogen bonds involving the carbonyl O acceptor atoms of the isonicotinamide ligands to the network. Finally, there are several short contacts indicative of weak C—H⋯S, C—H⋯O and C—H⋯N inter­actions. Numerical values of the hydrogen-bonding inter­actions are collated in Table 1 ▸.

## Database survey   

Some metal compounds based on isonicotinamide ligands and thio­cyanates anions are reported in the Cambridge Structure Database (Version 5.37, last update 2015; Groom *et al.*, 2016 ▸). Two Ni-clathrates, one with 9,10-anthra­quinone and the other with pyrene, in which Ni^II^ cations are connected by μ-1,3-bridging thio­cyanate ligands into coordination polymers (Sekiya *et al.*, 2009 ▸) and one very similar cadmium compound with 9,10-di­chloro­anthracene as clathrate mol­ecule (Sekiya & Nishikiori, 2005 ▸). Moreover, one compound comprising a three-dimensional coordination network based on Cd(SCN)_2_ (Yang *et al.*, 2001 ▸) and a compound built up of Cu–NCS layers are also reported (Đaković *et al.*, 2010 ▸). Very recently we reported two discrete complexes with isonicotinamide as co-ligand, one of which is based on Zn(NCS)_2_ with the Zn^II^ cation in tetra­hedral coordination (Neumann *et al.*, 2016*a* ▸) while the other is based on Co(NCS)_2_ in which the Co^II^ cation is octa­hedrally coordinated (Neumann *et al.*, 2016*b* ▸).

## Synthesis and crystallization   

Cobalt thio­cyanate and 4-isonicotinamide were obtained from Alfa Aesar and were used without any further purification. Crystals suitable for single crystal structure analysis were obtained from a mixture of 26.3 mg Co(NCS)_2_ (0.15 mmol) and 73.3 mg 4-isonicotinamide (0.6 mmol) in demineralized water (1.5 ml) within three days. The title compound could not be prepared as a single phase and was always contaminated with a second crystalline phase which could not be identified so far.

## Refinement   

Crystal data, data collection and structure refinement details are summarized in Table 2 ▸. The C—H and N—H hydrogen atoms were positioned in calculated positions with *U*_iso_(H) = 1.2*U*_eq_(C, N) using a riding model with C—H = 0.95 Å for aromatic and N—H = 0.88 Å for amide H atoms. The water hydrogen atoms were located in a difference map, and their bond lengths were constrained to O—H = 0.84 Å and with *U*_iso_(H) = 1.5*U*_eq_(O).

## Supplementary Material

Crystal structure: contains datablock(s) I, kw171. DOI: 10.1107/S2056989016012470/wm5313sup1.cif

Structure factors: contains datablock(s) I. DOI: 10.1107/S2056989016012470/wm5313Isup2.hkl

CCDC reference: 1497322

Additional supporting information: crystallographic information; 3D view; checkCIF report

## Figures and Tables

**Table 1:** Hydrogen-bond geometry (Å, °)

*D*—H⋯*A*	*D*—H	H⋯*A*	*D*⋯*A*	*D*—H⋯*A*
C11—H11⋯S2^i^	0.95	2.96	3.646 (2)	130
C14—H14⋯S1^ii^	0.95	2.84	3.785 (2)	172
C15—H15⋯O21^iii^	0.95	2.45	3.324 (3)	153
N12—H12*A*⋯O31^iv^	0.88	2.07	2.922 (2)	163
N12—H12*B*⋯S1^ii^	0.88	2.74	3.592 (2)	164
C21—H21⋯N2	0.95	2.65	3.245 (3)	122
C22—H22⋯O4	0.95	2.49	3.234 (2)	136
C24—H24⋯O2	0.95	2.58	3.423 (3)	149
C25—H25⋯N1	0.95	2.49	3.107 (2)	122
N22—H22*A*⋯S1^v^	0.88	2.79	3.6484 (18)	165
N22—H22*B*⋯O2	0.88	2.04	2.873 (2)	158
C32—H32⋯S2^iii^	0.95	3.00	3.826 (2)	146
N32—H32*A*⋯O2^vi^	0.88	2.25	3.121 (2)	172
N32—H32*B*⋯S2^iii^	0.88	2.57	3.4083 (19)	160
O1—H1*O*1⋯O21^iii^	0.84	1.96	2.7858 (19)	166
O1—H2*O*1⋯O21^vii^	0.84	2.01	2.8106 (19)	158
O2—H1*O*2⋯O3^viii^	0.84	1.83	2.650 (3)	164
O2—H2*O*2⋯N1^ix^	0.84	2.60	3.337 (2)	148
O2—H2*O*2⋯N31^ix^	0.84	2.66	3.363 (2)	143
O3—H1*O*3⋯O31	0.84	2.17	2.966 (3)	158
O3—H2*O*3⋯O4^x^	0.84	2.20	2.963 (2)	152
O4—H1*O*4⋯O11^xi^	0.84	1.91	2.723 (2)	163

Symmetry codes: (i) 
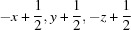
; (ii) 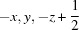; (iii) 
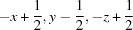
; (iv) 
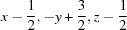
; (v) 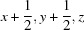; (vi) 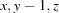; (vii) 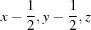; (viii) 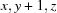; (ix) 
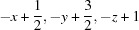
; (x) 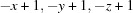; (xi) 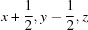.

**Table 2:** Experimental details

Crystal data	
Chemical formula	[Co(NCS)_2_(C_6_H_6_N_2_O)_3_(H_2_O)]·2.5H_2_O
*M*_r_	604.53
Crystal system, space group	Monoclinic, *C*2/*c*
Temperature (K)	200
*a*, *b*, *c* (Å)	19.2539 (16), 13.1442 (8), 20.7913 (16)
β (°)	97.327 (10)
*V* (Å^3^)	5218.8 (7)
*Z*	8
Radiation type	Mo *K*α
μ (mm^−1^)	0.87
Crystal size (mm)	0.11 × 0.08 × 0.06

Data collection	
Diffractometer	Stoe *IPDS2*
Absorption correction	Numerical (*X-SHAPE* and *X-RED32*; Stoe, 2008 ▸)
*T*_min_, *T*_max_	0.775, 0.920
No. of measured, independent and observed [*I* > 2σ(*I*)] reflections	29661, 6260, 5098
*R*_int_	0.060
(sin θ/λ)_max_ (Å^−1^)	0.661

Refinement	
*R*[*F* ^2^ > 2σ(*F* ^2^)], *wR*(*F* ^2^), *S*	0.040, 0.094, 1.01
No. of reflections	6260
No. of parameters	339
H-atom treatment	H-atom parameters constrained
Δρ_max_, Δρ_min_ (e Å^−3^)	0.33, −0.55

Computer programs: *X-AREA* (Stoe, 2008 ▸), *SHELXS97* (Sheldrick, 2008 ▸), *SHELXL2014* (Sheldrick, 2015 ▸), *XP* in *SHELXTL* (Sheldrick, 2008 ▸), *DIAMOND* (Brandenburg, 1999 ▸) and *publCIF* (Westrip, 2010 ▸).

## supplementary crystallographic information

### Crystal data

**Table:**

[Co(NCS)_2_(C_6_H_6_N_2_O)_3_(H_2_O)]·2.5H_2_O	*F*(000) = 2496
*M**_r_* = 604.53	*D*_x_ = 1.539 Mg m^−^^3^
Monoclinic, *C*2/*c*	Mo *K*α radiation, λ = 0.71073 Å
*a* = 19.2539 (16) Å	Cell parameters from 6260 reflections
*b* = 13.1442 (8) Å	θ = 4.3–56°
*c* = 20.7913 (16) Å	µ = 0.87 mm^−^^1^
β = 97.327 (10)°	*T* = 200 K
*V* = 5218.8 (7) Å^3^	Block, red-brown
*Z* = 8	0.11 × 0.08 × 0.06 mm

### Data collection

**Table:**

Stoe IPDS-2 diffractometer	5098 reflections with *I* > 2σ(*I*)
ω–scans	*R*_int_ = 0.060
Absorption correction: numerical (X-SHAPE and X-RED32; Stoe, 2008)	θ_max_ = 28.0°, θ_min_ = 2.6°
*T*_min_ = 0.775, *T*_max_ = 0.920	*h* = −25→25
29661 measured reflections	*k* = −17→17
6260 independent reflections	*l* = −27→27

### Refinement

**Table:**

Refinement on *F*^2^	0 restraints
Least-squares matrix: full	Hydrogen site location: mixed
*R*[*F*^2^ > 2σ(*F*^2^)] = 0.040	H-atom parameters constrained
*wR*(*F*^2^) = 0.094	*w* = 1/[σ^2^(*F*_o_^2^) + (0.0525*P*)^2^ + 5.523*P*] where *P* = (*F*_o_^2^ + 2*F*_c_^2^)/3
*S* = 1.01	(Δ/σ)_max_ = 0.001
6260 reflections	Δρ_max_ = 0.33 e Å^−^^3^
339 parameters	Δρ_min_ = −0.55 e Å^−^^3^

### Special details

**Table:**

Geometry. All esds (except the esd in the dihedral angle between two l.s. planes) are estimated using the full covariance matrix. The cell esds are taken into account individually in the estimation of esds in distances, angles and torsion angles; correlations between esds in cell parameters are only used when they are defined by crystal symmetry. An approximate (isotropic) treatment of cell esds is used for estimating esds involving l.s. planes.

### Fractional atomic coordinates and isotropic or equivalent isotropic displacement parameters (Å^2^)

**Table:**

	*x*	*y*	*z*	*U*_iso_*/*U*_eq_	
Co1	0.18635 (2)	0.67227 (2)	0.31272 (2)	0.01581 (7)	
N1	0.14313 (9)	0.70880 (14)	0.39620 (8)	0.0246 (3)	
C1	0.10950 (9)	0.72960 (14)	0.43682 (9)	0.0186 (3)	
S1	0.06154 (3)	0.75764 (4)	0.49356 (3)	0.02889 (12)	
N2	0.22147 (9)	0.62103 (13)	0.22769 (8)	0.0233 (3)	
C2	0.25664 (10)	0.61371 (14)	0.18638 (9)	0.0201 (4)	
S2	0.30765 (3)	0.60830 (4)	0.12936 (3)	0.02793 (12)	
N11	0.12709 (8)	0.79048 (12)	0.25649 (8)	0.0209 (3)	
C11	0.12791 (11)	0.88801 (16)	0.27443 (10)	0.0262 (4)	
H11	0.1524	0.9059	0.3155	0.031*	
C12	0.09489 (11)	0.96437 (16)	0.23626 (10)	0.0269 (4)	
H12	0.0975	1.0329	0.2509	0.032*	
C13	0.05785 (10)	0.94012 (15)	0.17636 (9)	0.0206 (4)	
C14	0.05573 (11)	0.83848 (16)	0.15789 (10)	0.0264 (4)	
H14	0.0305	0.8182	0.1177	0.032*	
C15	0.09093 (11)	0.76706 (15)	0.19885 (10)	0.0263 (4)	
H15	0.0894	0.6979	0.1855	0.032*	
C16	0.02360 (11)	1.02471 (16)	0.13489 (10)	0.0250 (4)	
N12	−0.01376 (10)	0.99985 (14)	0.07899 (9)	0.0324 (4)	
H12A	−0.0341	1.0476	0.0536	0.039*	
H12B	−0.0182	0.9356	0.0673	0.039*	
O11	0.03115 (10)	1.11340 (12)	0.15299 (8)	0.0415 (4)	
N21	0.27358 (8)	0.77939 (12)	0.33884 (8)	0.0187 (3)	
C21	0.32723 (10)	0.78644 (16)	0.30338 (10)	0.0246 (4)	
H21	0.3289	0.7402	0.2685	0.030*	
C22	0.38017 (10)	0.85805 (16)	0.31536 (10)	0.0235 (4)	
H22	0.4164	0.8613	0.2884	0.028*	
C23	0.37979 (9)	0.92477 (14)	0.36683 (9)	0.0174 (3)	
C24	0.32564 (10)	0.91704 (15)	0.40465 (9)	0.0207 (4)	
H24	0.3239	0.9609	0.4407	0.025*	
C25	0.27411 (10)	0.84428 (15)	0.38891 (9)	0.0212 (4)	
H25	0.2371	0.8400	0.4150	0.025*	
C26	0.43638 (9)	1.00411 (14)	0.37791 (9)	0.0187 (4)	
N22	0.45023 (9)	1.04280 (14)	0.43691 (8)	0.0252 (4)	
H22A	0.4825	1.0902	0.4449	0.030*	
H22B	0.4272	1.0212	0.4682	0.030*	
O21	0.46730 (7)	1.03177 (11)	0.33192 (7)	0.0231 (3)	
N31	0.24699 (8)	0.55232 (12)	0.36533 (7)	0.0182 (3)	
C31	0.24100 (10)	0.45608 (15)	0.34455 (9)	0.0222 (4)	
H31	0.2135	0.4428	0.3042	0.027*	
C32	0.27278 (10)	0.37491 (15)	0.37887 (10)	0.0235 (4)	
H32	0.2667	0.3076	0.3625	0.028*	
C33	0.31380 (9)	0.39326 (14)	0.43780 (9)	0.0183 (3)	
C34	0.32139 (10)	0.49318 (15)	0.45905 (10)	0.0238 (4)	
H34	0.3497	0.5088	0.4986	0.029*	
C35	0.28715 (11)	0.56976 (15)	0.42182 (10)	0.0245 (4)	
H35	0.2924	0.6378	0.4370	0.029*	
C36	0.35105 (10)	0.31096 (15)	0.47912 (9)	0.0203 (4)	
N32	0.32523 (10)	0.21708 (13)	0.47146 (9)	0.0283 (4)	
H32A	0.3452	0.1668	0.4949	0.034*	
H32B	0.2882	0.2054	0.4430	0.034*	
O31	0.40296 (7)	0.33185 (11)	0.51840 (7)	0.0257 (3)	
O1	0.09982 (7)	0.56631 (11)	0.29295 (7)	0.0226 (3)	
H1O1	0.0844	0.5474	0.2552	0.034*	
H2O1	0.0639	0.5683	0.3120	0.034*	
O2	0.38171 (8)	1.02681 (13)	0.55143 (7)	0.0324 (3)	
H1O2	0.4007	1.0670	0.5798	0.049*	
H2O2	0.3617	0.9813	0.5707	0.049*	
O3	0.43909 (14)	0.17955 (15)	0.62254 (12)	0.0672 (7)	
H1O3	0.4391	0.2301	0.5978	0.101*	
H2O3	0.4411	0.2036	0.6602	0.101*	
O4	0.5000	0.73797 (19)	0.2500	0.0451 (6)	
H1O4	0.5181	0.7008	0.2238	0.068*	

### Atomic displacement parameters (Å^2^)

**Table:**

	*U*^11^	*U*^22^	*U*^33^	*U*^12^	*U*^13^	*U*^23^
Co1	0.01664 (12)	0.01500 (12)	0.01570 (12)	−0.00072 (9)	0.00174 (9)	0.00114 (9)
N1	0.0253 (8)	0.0258 (9)	0.0237 (8)	−0.0016 (7)	0.0075 (7)	−0.0018 (7)
C1	0.0190 (8)	0.0162 (8)	0.0196 (8)	−0.0020 (7)	−0.0014 (7)	0.0010 (7)
S1	0.0318 (3)	0.0333 (3)	0.0232 (2)	0.0063 (2)	0.0099 (2)	−0.0006 (2)
N2	0.0264 (8)	0.0240 (9)	0.0200 (8)	−0.0008 (7)	0.0048 (6)	−0.0017 (6)
C2	0.0245 (9)	0.0144 (8)	0.0202 (9)	0.0006 (7)	−0.0012 (7)	0.0016 (7)
S2	0.0301 (3)	0.0310 (3)	0.0243 (2)	0.0070 (2)	0.0096 (2)	0.0061 (2)
N11	0.0225 (8)	0.0187 (8)	0.0205 (8)	0.0005 (6)	−0.0010 (6)	0.0020 (6)
C11	0.0303 (10)	0.0226 (10)	0.0230 (9)	0.0048 (8)	−0.0067 (8)	−0.0034 (8)
C12	0.0331 (10)	0.0188 (9)	0.0267 (10)	0.0045 (8)	−0.0044 (8)	−0.0048 (8)
C13	0.0208 (9)	0.0199 (9)	0.0205 (9)	0.0029 (7)	0.0009 (7)	0.0016 (7)
C14	0.0316 (10)	0.0222 (10)	0.0222 (9)	0.0012 (8)	−0.0084 (8)	0.0000 (8)
C15	0.0342 (10)	0.0158 (9)	0.0259 (10)	−0.0007 (8)	−0.0083 (8)	0.0000 (8)
C16	0.0282 (10)	0.0209 (10)	0.0256 (10)	0.0060 (8)	0.0020 (8)	0.0015 (8)
N12	0.0417 (10)	0.0221 (9)	0.0294 (9)	0.0076 (8)	−0.0105 (8)	0.0027 (7)
O11	0.0673 (12)	0.0199 (8)	0.0337 (9)	0.0107 (8)	−0.0072 (8)	−0.0019 (7)
N21	0.0172 (7)	0.0158 (7)	0.0227 (8)	−0.0023 (6)	0.0012 (6)	0.0014 (6)
C21	0.0255 (9)	0.0214 (10)	0.0279 (10)	−0.0026 (8)	0.0071 (8)	−0.0058 (8)
C22	0.0221 (9)	0.0225 (10)	0.0273 (10)	−0.0032 (7)	0.0083 (7)	−0.0022 (8)
C23	0.0160 (8)	0.0147 (8)	0.0209 (9)	−0.0002 (6)	0.0000 (6)	0.0041 (7)
C24	0.0204 (8)	0.0212 (9)	0.0205 (9)	−0.0032 (7)	0.0027 (7)	−0.0025 (7)
C25	0.0189 (8)	0.0222 (10)	0.0228 (9)	−0.0041 (7)	0.0041 (7)	−0.0011 (7)
C26	0.0163 (8)	0.0171 (9)	0.0223 (9)	0.0011 (7)	0.0010 (7)	0.0027 (7)
N22	0.0237 (8)	0.0284 (9)	0.0235 (8)	−0.0098 (7)	0.0031 (6)	−0.0026 (7)
O21	0.0214 (6)	0.0260 (7)	0.0220 (7)	−0.0043 (5)	0.0026 (5)	0.0057 (6)
N31	0.0199 (7)	0.0159 (7)	0.0185 (7)	0.0005 (6)	0.0007 (6)	0.0015 (6)
C31	0.0256 (9)	0.0195 (9)	0.0197 (9)	0.0016 (7)	−0.0043 (7)	−0.0037 (7)
C32	0.0292 (10)	0.0159 (9)	0.0239 (9)	−0.0003 (7)	−0.0024 (8)	−0.0017 (7)
C33	0.0178 (8)	0.0163 (9)	0.0206 (9)	0.0008 (6)	0.0016 (7)	0.0008 (7)
C34	0.0270 (9)	0.0190 (9)	0.0231 (9)	0.0007 (7)	−0.0058 (7)	−0.0013 (7)
C35	0.0297 (10)	0.0160 (9)	0.0252 (10)	−0.0007 (7)	−0.0068 (8)	−0.0011 (7)
C36	0.0212 (8)	0.0189 (9)	0.0209 (9)	0.0032 (7)	0.0026 (7)	−0.0009 (7)
N32	0.0318 (9)	0.0162 (8)	0.0337 (10)	0.0011 (7)	−0.0085 (7)	0.0036 (7)
O31	0.0254 (7)	0.0227 (7)	0.0267 (7)	0.0016 (6)	−0.0057 (6)	−0.0004 (6)
O1	0.0187 (6)	0.0271 (7)	0.0220 (7)	−0.0063 (5)	0.0023 (5)	−0.0021 (6)
O2	0.0374 (8)	0.0337 (9)	0.0272 (8)	−0.0031 (7)	0.0085 (6)	0.0033 (7)
O3	0.1016 (18)	0.0296 (10)	0.0598 (14)	−0.0097 (11)	−0.0299 (13)	0.0075 (9)
O4	0.0690 (18)	0.0284 (13)	0.0415 (14)	0.000	0.0211 (13)	0.000

### Geometric parameters (Å, º)

**Table:**

Co1—N1	2.0746 (17)	C23—C24	1.387 (3)
Co1—N2	2.0834 (17)	C23—C26	1.505 (2)
Co1—O1	2.1703 (13)	C24—C25	1.387 (3)
Co1—N31	2.1725 (16)	C24—H24	0.9500
Co1—N11	2.1778 (16)	C25—H25	0.9500
Co1—N21	2.2059 (15)	C26—O21	1.244 (2)
N1—C1	1.161 (3)	C26—N22	1.323 (3)
C1—S1	1.6304 (19)	N22—H22A	0.8800
N2—C2	1.164 (3)	N22—H22B	0.8800
C2—S2	1.635 (2)	N31—C31	1.337 (2)
N11—C11	1.335 (3)	N31—C35	1.341 (2)
N11—C15	1.343 (3)	C31—C32	1.382 (3)
C11—C12	1.383 (3)	C31—H31	0.9500
C11—H11	0.9500	C32—C33	1.392 (3)
C12—C13	1.391 (3)	C32—H32	0.9500
C12—H12	0.9500	C33—C34	1.387 (3)
C13—C14	1.389 (3)	C33—C36	1.505 (3)
C13—C16	1.506 (3)	C34—C35	1.384 (3)
C14—C15	1.385 (3)	C34—H34	0.9500
C14—H14	0.9500	C35—H35	0.9500
C15—H15	0.9500	C36—O31	1.238 (2)
C16—O11	1.228 (3)	C36—N32	1.332 (3)
C16—N12	1.327 (3)	N32—H32A	0.8800
N12—H12A	0.8800	N32—H32B	0.8800
N12—H12B	0.8800	O1—H1O1	0.8401
N21—C25	1.345 (2)	O1—H2O1	0.8398
N21—C21	1.347 (2)	O2—H1O2	0.8401
C21—C22	1.386 (3)	O2—H2O2	0.8400
C21—H21	0.9500	O3—H1O3	0.8400
C22—C23	1.384 (3)	O3—H2O3	0.8400
C22—H22	0.9500	O4—H1O4	0.8400
			
N1—Co1—N2	173.17 (7)	C22—C21—H21	118.5
N1—Co1—O1	85.81 (6)	C23—C22—C21	119.53 (18)
N2—Co1—O1	87.49 (6)	C23—C22—H22	120.2
N1—Co1—N31	89.65 (6)	C21—C22—H22	120.2
N2—Co1—N31	88.90 (6)	C22—C23—C24	118.04 (17)
O1—Co1—N31	88.85 (6)	C22—C23—C26	118.85 (16)
N1—Co1—N11	92.60 (7)	C24—C23—C26	123.08 (17)
N2—Co1—N11	88.83 (7)	C25—C24—C23	118.91 (18)
O1—Co1—N11	91.10 (6)	C25—C24—H24	120.5
N31—Co1—N11	177.74 (6)	C23—C24—H24	120.5
N1—Co1—N21	91.16 (6)	N21—C25—C24	123.68 (17)
N2—Co1—N21	95.51 (6)	N21—C25—H25	118.2
O1—Co1—N21	176.67 (6)	C24—C25—H25	118.2
N31—Co1—N21	89.76 (6)	O21—C26—N22	122.75 (17)
N11—Co1—N21	90.41 (6)	O21—C26—C23	119.55 (17)
C1—N1—Co1	169.72 (16)	N22—C26—C23	117.69 (16)
N1—C1—S1	179.25 (19)	C26—N22—H22A	120.0
C2—N2—Co1	159.31 (16)	C26—N22—H22B	120.0
N2—C2—S2	177.44 (19)	H22A—N22—H22B	120.0
C11—N11—C15	117.13 (17)	C31—N31—C35	117.46 (16)
C11—N11—Co1	123.26 (13)	C31—N31—Co1	120.35 (12)
C15—N11—Co1	119.48 (13)	C35—N31—Co1	121.99 (13)
N11—C11—C12	123.26 (18)	N31—C31—C32	123.31 (17)
N11—C11—H11	118.4	N31—C31—H31	118.3
C12—C11—H11	118.4	C32—C31—H31	118.3
C11—C12—C13	119.55 (19)	C31—C32—C33	119.01 (18)
C11—C12—H12	120.2	C31—C32—H32	120.5
C13—C12—H12	120.2	C33—C32—H32	120.5
C14—C13—C12	117.48 (18)	C34—C33—C32	117.99 (17)
C14—C13—C16	123.79 (18)	C34—C33—C36	118.40 (17)
C12—C13—C16	118.71 (18)	C32—C33—C36	123.60 (17)
C15—C14—C13	119.15 (18)	C35—C34—C33	119.14 (18)
C15—C14—H14	120.4	C35—C34—H34	120.4
C13—C14—H14	120.4	C33—C34—H34	120.4
N11—C15—C14	123.41 (19)	N31—C35—C34	123.07 (18)
N11—C15—H15	118.3	N31—C35—H35	118.5
C14—C15—H15	118.3	C34—C35—H35	118.5
O11—C16—N12	122.13 (19)	O31—C36—N32	122.80 (18)
O11—C16—C13	119.94 (19)	O31—C36—C33	120.18 (17)
N12—C16—C13	117.93 (18)	N32—C36—C33	117.02 (17)
C16—N12—H12A	120.0	C36—N32—H32A	120.0
C16—N12—H12B	120.0	C36—N32—H32B	120.0
H12A—N12—H12B	120.0	H32A—N32—H32B	120.0
C25—N21—C21	116.72 (16)	Co1—O1—H1O1	122.5
C25—N21—Co1	121.72 (12)	Co1—O1—H2O1	123.2
C21—N21—Co1	121.48 (13)	H1O1—O1—H2O1	103.7
N21—C21—C22	123.09 (18)	H1O2—O2—H2O2	107.4
N21—C21—H21	118.5	H1O3—O3—H2O3	105.6

### Hydrogen-bond geometry (Å, º)

**Table:**

*D*—H···*A*	*D*—H	H···*A*	*D*···*A*	*D*—H···*A*
C11—H11···S2^i^	0.95	2.96	3.646 (2)	130
C14—H14···S1^ii^	0.95	2.84	3.785 (2)	172
C15—H15···O21^iii^	0.95	2.45	3.324 (3)	153
N12—H12*A*···O31^iv^	0.88	2.07	2.922 (2)	163
N12—H12*B*···S1^ii^	0.88	2.74	3.592 (2)	164
C21—H21···N2	0.95	2.65	3.245 (3)	122
C22—H22···O4	0.95	2.49	3.234 (2)	136
C24—H24···O2	0.95	2.58	3.423 (3)	149
C25—H25···N1	0.95	2.49	3.107 (2)	122
N22—H22*A*···S1^v^	0.88	2.79	3.6484 (18)	165
N22—H22*B*···O2	0.88	2.04	2.873 (2)	158
C32—H32···S2^iii^	0.95	3.00	3.826 (2)	146
N32—H32*A*···O2^vi^	0.88	2.25	3.121 (2)	172
N32—H32*B*···S2^iii^	0.88	2.57	3.4083 (19)	160
O1—H1*O*1···O21^iii^	0.84	1.96	2.7858 (19)	166
O1—H2*O*1···O21^vii^	0.84	2.01	2.8106 (19)	158
O2—H1*O*2···O3^viii^	0.84	1.83	2.650 (3)	164
O2—H2*O*2···N1^ix^	0.84	2.60	3.337 (2)	148
O2—H2*O*2···N31^ix^	0.84	2.66	3.363 (2)	143
O3—H1*O*3···O31	0.84	2.17	2.966 (3)	158
O3—H2*O*3···O4^x^	0.84	2.20	2.963 (2)	152
O4—H1*O*4···O11^xi^	0.84	1.91	2.723 (2)	163

Symmetry codes: (i) −*x*+1/2, *y*+1/2, −*z*+1/2; (ii) −*x*, *y*, −*z*+1/2; (iii) −*x*+1/2, *y*−1/2, −*z*+1/2; (iv) *x*−1/2, −*y*+3/2, *z*−1/2; (v) *x*+1/2, *y*+1/2, *z*; (vi) *x*, *y*−1, *z*; (vii) *x*−1/2, *y*−1/2, *z*; (viii) *x*, *y*+1, *z*; (ix) −*x*+1/2, −*y*+3/2, −*z*+1; (x) −*x*+1, −*y*+1, −*z*+1; (xi) *x*+1/2, *y*−1/2, *z*.
